# Intraoperative precision of 25-gauge beveled-tip versus 23-gauge flat-tip probes in day surgery vitrectomy for proliferative diabetic retinopathy: a comparative cohort study

**DOI:** 10.1186/s40942-025-00746-6

**Published:** 2025-11-05

**Authors:** Daxi Xue, Yanchun Zhang, Ziwei Kang, Jiamin Zheng, Yingnan He, Xin Luo

**Affiliations:** Shaanxi Eye Hospital, Xi’an People’s Hospital (Xi’an Fourth Hospital), Affiliated People’s Hospital of Northwest University, No. 21 Jiefang Road, Xi’an, Shaanxi 710004 China

**Keywords:** Proliferative diabetic retinopathy, Vitrectomy, 25-gauge, Beveled-tip probe, Intraoperative bleeding, Endodiathermy, Day surgery

## Abstract

**Background:**

To compare the intraoperative performance and safety of the 10k cuts-per-minute (cpm)/25-gauge (G) beveled-tip probe (BTP) versus the 5k-cpm/23-G flat-tip probe (FTP) in microincision vitrectomy surgery (MIVS) for proliferative diabetic retinopathy (PDR).

**Methods:**

This comparative interventional cohort study enrolled 173 eyes of 156 consecutive patients with PDR. Eyes underwent MIVS by a single surgeon from April 2022 to June 2023, utilizing either the 10k/25-G BTP (study group) or the 5k/23-G FTP (control group). All patients completed 3-month protocolized follow-up; 72.8% (126 eyes) had extended observational tracking to 6 months. Intraoperative parameters (instrument exchange, endodiathermy application), surgical times, and postoperative outcomes were analyzed. Main Outcome Measures: Rate of instrument exchange, number of endodiathermy applications, and operative time.

**Results:**

Baseline demographics and operative characteristics were comparable between groups. Total operative time (63.30 ± 18.38 vs. 59.84 ± 19.28 min, *P* = 0.14) and vitrectomy time (36.55 ± 15.54 vs. 35.59 ± 16.59, *P* = 0.67) did not differ significantly. The 25-G BTP group demonstrated a 41% reduction in instrument exchanges (1.31 ± 2.02 vs. 2.22 ± 2.71, *P* = 0.003) and required 58% fewer endodiathermy applications (2.28 ± 3.33 vs. 5.49 ± 6.49 sites, *P* < 0.0001). Sutureless sclerotomy rates were higher in the 25-G group (76.7% vs. 16.1%, *P* < 0.0001). Postoperative best-corrected visual acuity improvement, complication rates, and intraocular pressure control were equivalent between groups at 3 months and 6 months.

**Conclusions:**

While both systems achieve equivalent visual and anatomical outcomes in PDR surgery, the 10k/25-G BTP system facilitates a novel “*Trim and Excision*” Technique, enabling more precise dissection of fibrovascular tissue. This technique translates to superior intraoperative efficiency and safety, significantly reducing instrument dependence and iatrogenic hemorrhage without compromising surgical speed or postoperative recovery.

**Clinical trial registration:**

This trial is registered with the Chinese Clinical Trial Registry (http://www.chictr.org.cn, registration number ChiCTR2300067743). Date of registration: 2023-01-20.

**Supplementary Information:**

The online version contains supplementary material available at 10.1186/s40942-025-00746-6.

## Background

Proliferative diabetic retinopathy (PDR) is a severe condition that can cause significant vision loss without timely treatment. Pars plana vitrectomy (PPV) is the standard care for a variety of complications, including chronic vitreous haemorrhage (VH), fibrovascular proliferation (FVP) with tractional or rhegmatogenous retinal detachment (RD). It is also used to treat taut posterior hyaloid traction contributing to nonresolving macular edema or macular hole, as well as neovascular glaucoma (NVG) requiring intraocular laser intervention [1]. Therefore, releasing vitreous traction and managing fibrovascular membranes played a critical role in diabetic surgery.

Surgeons primarily address these membranes through segmentation or delamination techniques. Although 23-G instruments are generally favored for their stiffness and maneuverability in most cases [2], they may be risky when membranes tightly adhere to the retina. Exploring more effective and safer methods to manage vitreoretinal adhesions between the retina and fibrovascular proliferation is crucial. Smaller-gauge vitrectomy systems like 25-G offer advantages for precise delamination [3, 4]. Enhanced designs, such as beveled-tip 25-G probes with cutting rates of 10,000 cuts per minute (cpm) (e.g., Advanced ULTRAVIT^®^), have been developed. These innovations promise improved efficiency and safety in minimally invasive vitrectomy surgery (MIVS) [5, 6].

However, there is limited comparative evidence on intraoperative precision of 10k/25-G beveled-tip probes (BTP) versus 5k/23-G flat-tip probes (FTP) in PDR day surgery. To address this gap, a cohort study was conducted to compare these probes in PDR patients. The objective of this study was to evaluate the intraoperative precision and efficiency, as well as the postoperative outcomes of the surgical intervention.

## Methods

### Study design and population

This cohort study analyzed 173 consecutive eyes from 156 patients (69 females, 87 males) who underwent primary PPV for PDR in day surgery from April 2022 to June 2023. All surgical procedures were carried out by a single surgeon, Dr. YC Zhang. Ethics approval was obtained from the Ethics Committee of Xi’an People’s Hospital (Declaration of Helsinki). Written consent was obtained from all participants.

### Inclusion and exclusion criteria

Patients with Type II diabetes and PDR complications were included if they had any of the following: recurrent or persistent VH, dense macular subhyaloid hemorrhage, progressive FVP threatening macula, tractional or rhegmatogenous RD, iris erythema or NVG. Exclusion Criteria included: (1) Prior vitreoretinal surgery; (2) Other retinal or choroidal disorders (e.g., uveitis, age-related macular degeneration [AMD], primary rhegmatogenous RD, retinal vascular occlusion); (3) Baseline hypotony (intraocular pressure [IOP] < 6mmHg); (4) Primary glaucoma; and (5) Uncontrolled systemic disease.

### Sample size justification and cohort assembly

#### Phase 1: Primary 3-month study

The sample size for the prospective main study was calculated using a power analysis (PASS 15.0, NCSS LLC) based on preliminary data from a pilot study of 16 matched patients (8 per technique). The primary endpoint was the number of intraoperative electrocoagulation sites. The mean number of coagulation sites observed was 2.13 ± 3.31 for BTP and 5.00 ± 5.10 for FTP; the mean difference was 2.87. To achieve 80% power with a two-sided α of 0.05 (Satterthwaite t-test), 37 patients per group were required. Accounting for an estimated 10% attrition rate, the target sample size for the prospective BTP group was set at 42 eyes. The control group (FTP) was a retrospective cohort that was matched to the BTP cases at approximately a 1:3 ratio based on FVP grade, yielding an initial 111 eyes.

**Fig. 1:**
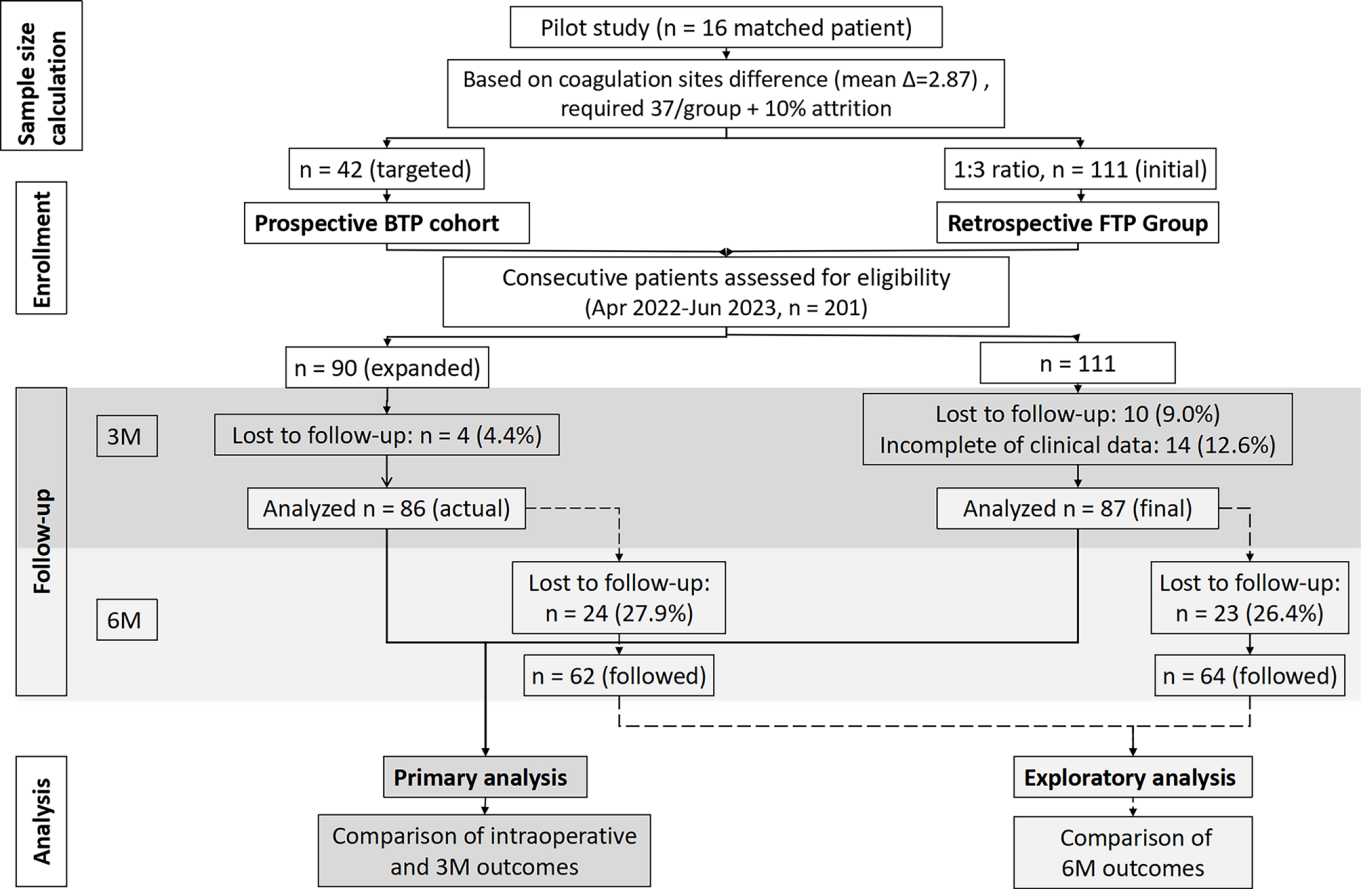
Participant flow diagram. The study was conducted in two phases: (1) the primary prospective phase for intraoperative and 3-month outcomes, and (2) an exploratory phase for extended 6-month outcomes in a subset of patients. Abbreviations: BTP, beveled-tip probe; FTP, flat-tip probe

To assemble the cohorts for this primary comparison, the FTP group was selected from patients who underwent surgery between April 2022 and January 2023. The BTP group was consecutively enrolled from January to June 2023. Participants with both eyes affected were allowed to enroll two eyes in the study. After applying inclusion and exclusion criteria and accounting for loss to follow-up or incomplete clinical data, the final analytical cohorts for the primary 3-month analysis consisted of 87 eyes in the FTP group, while the BTP group sample size was expanded to 86 eyes due to availability of clinical resources, as shown in Fig. 1.

#### Phase 2: Extended follow-up analysis (exploratory 6-month outcomes)

Subsequent to the primary analysis, we retrospectively identified a subset of patients from both the original BTP and FTP cohorts who had completed 6-month follow-up. This post-hoc exploratory analysis aimed to evaluate longer-term safety and functional outcomes. The final extended cohort for the 6-month analysis included 62 eyes (BTP) and 64 eyes (FTP).

### Preoperative and postoperative assessments

Prior to undergoing surgery, all patients underwent comprehensive ophthalmic examinations. These included refraction (KR-8900, Topcon, Japan), best-corrected visual acuity (BCVA), slit-lamp biomicroscopy, IOP, dilated fundus examination, swept-source optical coherence tomography (SS-OCT; DRI-OCT, Topcon, Japan), and B-scan ultrasonography. Postoperative follow-up was processed on postoperative day 1, week 2, and month 1, 2, 3, and 6. The assessments encompassed BCVA, IOP, slit-lamp biomicroscopy, and dilated fundus examination. Additionally, SS-OCT and B-scan ultrasonography were repeated as clinically indicated. The following complications were documented: hypotony (IOP ≤ 6 mmHg), ocular hypertension (IOP ≥ 25 mmHg) [7], RD, endophthalmitis, and choroidal detachment.

Baseline demographic data, including age, gender, previous history of laser coagulation and anti-VEGF treatment, type of diabetes, and systemic features such as glycosylated hemoglobin (HbA1c), systolic arterial pressure (SAP), diastolic arterial pressure (DAP), and estimated glomerular filtration rate (eGFR) and serum creatinine (Scr), were collected.

### Clinical procedures

Before surgery, the surgeon evaluated eyes that had not received an intravitreal anti-VEGF injection within the past month. This evaluation involved a careful analysis to ascertain whether the recommendation for the administration of adjuvant anti-VEGF agents was deemed necessary. The criteria for preoperative anti-VEGF injection were as follows: active FVP (if it contained visible neovascularization tissue or was associated with any degree of VH) or massive fresh vitreous haemorrhage. The anti-VEGF drug, including aflibercept (Eylea; Bayer, Leverkusen, Germany), ranibizumab (Lucentis; Genentech, Inc., South San Francisco, CA), or conbercept (KH902; Chengdu Kanghong Biotech Co., Ltd., Sichuan, China), was administered 3–5 days prior to PPV, according to the patients’ preferences.

Vitrectomy was performed in day surgery under retrobulbar anaesthesia or general anaesthesia. The procedure used a Constellation platform (Alcon, Fort Worth, TX). The FTP group was operated with 23-G, 5,000 cpm, 0-550 mmHg linear aspiration, while the BTP group used 25-G, 10,000 cpm and 0-650 mmHg linear aspiration. Intraocular pressure control setting on the Alcon Constellation device was turned on throughout the surgical procedure. All patients underwent standard vitrectomy techniques to remove the peripheral vitreous and proliferative membranes. Segmentation, delamination, lift and shave [4], bimanual, and viscodissection techniques were used when necessary. The IOP was set at 26 mmHg in all cases and was raised to 60 mmHg for about 1 to 3 min if bleeding occurred during the vitrectomy. If bleeding persisted and was active, intraocular bipolar electrocoagulation was used. Furthermore, endo-laser pan-retinal photocoagulation (PRP) was performed in all eyes during PPV. Intraocular tamponade was performed using sterile air, gas (9% perfluoropropane, C3F8; Alcon Laboratories), or silicone oil, if necessary. At the end of the operation, 0.05 ml triamcinolone acetonide was injected into the vitreous. After removing each cannula, the sclerotomy site was compressed with forceps to close the wound. When the sclerotomy was leaking or tamponade with silicone oil was used, the wound was closed with 8 − 0 Vicryl suture. Simultaneous cataract phacoemulsification surgery was conducted when the lens’ opacity obstructed fundus observation or in patients aged 60 years or older. Operation videos recorded the intraoperative data. Operation time was defined as the time to perform the whole surgical procedure, including specific procedures such as PRP, tamponade agent placement, and incision closure. Actual vitrectomy time was defined as the duration required to complete core and peripheral vitrectomy and full processing of fibrovascular membrane. It started from the first insertion of the cutter and ended with the last extraction of the cutter from the port for vitrectomy.

### Severity grading at baseline

The “complexity score” (CS) and the FVP grade were utilized to assess the severity of the PDR. The CS was quantified based on the number of quadrants involved and the location of FVP. It also considered the presence of tractional retinal detachment (TRD), TRD with rhegmatogenous, and posterior vitreous detachment (PVD) in the macular area [8]. The extent of FVP was classified into four grades, according to the severity of vitreoretinal adhesion: Grade 1: Multiple focal adhesions with or without plaque-like broad adhesion at a single site; Grade 2: Broad adhesions at more than one but fewer than three sites located posterior to the equator; Grade 3: Broad adhesions at more than three sites, located posterior to the equator or extending beyond the equator within one quadrant; Grade 4: Broad adhesions extending beyond the equator for more than one quadrant [9].

### Outcome measures

The primary outcome measures were intraoperative metrics, including the number of electrocoagulated bleeding sites and instrument exchanges, total operation time, and vitrectomy time. The secondary outcome measures include: the type of tamponade employed, the occurrence of iatrogenic retinal tears, BCVA, and IOP. Postoperative complications were also analysed, including hypotony (IOP ≤ 6 mmHg), hypertension (IOP ≥ 25 mmHg), RD, endophthalmitis, recurrence of VH or fibro-membrane.

### Statistics analyses

Statistical analyses were performed using SPSS version 21 (SPSS, Inc., Chicago, IL) and GraphPad Prism 10 (GraphPad Software, LLC.). Descriptive statistics were presented as mean ± SD or percentages. Chi-squared test or Mann–Whitney test was used to compare categorical or continuous variables between two groups. Paired t-tests were applied for intra-group changes. Furthermore, multivariable analyses were performed using linear regression (for continuous outcomes) or logistic regression (for binary outcomes), adjusted for clinically relevant confounders, including demographics (age, sex), systemic factors (HbA1c, diabetes duration, eGFR, Scr), ocular factors (baseline disease severity: FVP grade, CS, BCVA; pre-treatment: prior retinal photocoagulation, adjuvant anti-VEGF agents), and surgical factors (phacovitrectomy, tamponade type). We retrospectively collected extended cohort data from 126 patients who completed 6-month follow-up (FTP group: 64; BTP group: 62). A post-hoc exploratory analysis was then performed, focusing on late outcomes and complications. P-values less than 0.05 were considered statistically significant.

## Result

### Baseline patient characteristics

This study included 173 eyes from 156 patients. Baseline demographics, systemic comorbidities, ocular characteristics, and previous treatment history were comparable between the 23-G FTP and 25-G BTP groups. Details are provided in Tables 1A, 1B, and 1C.

No statistically significant differences existed in key prognostic factors, including age, diabetes duration, prevalence of hypertension, HbA1c level, renal function (eGFR), baseline visual acuity (BCVA), or intraocular pressure (IOP) (all *P* > 0.05, Tables 1A and 1B).

Disease severity was comparable between groups, as measured by the CS and FVP grade (Table 1B). The distribution of FVP grades (1–4) and the mean CS showed no significant intergroup differences (all *P* > 0.05). Although not statistically significant, there was a higher proportion of severe (Grade 3/4) cases in the 25-G BTP group (59.3% vs. 51.7%, *P* = 0.78).

Previous treatment histories of the groups were similar (Table 1C), with equivalent rates of prior retinal photocoagulation and anti-VEGF injections performed more than one month before surgery. Crucially, the proportion of eyes receiving preoperative anti-VEGF injections within the critical one-month window before vitrectomy did not differ significantly between groups. Similarly, the choice of drug (aflibercept, ranibizumab, or conbercept) and the mean interval between injection and surgery were comparable (all *P* > 0.05).

**Table 1A:** Baseline demographics and systemic features

	23-G	25-G	*P*
No. [eyes (patients)]	87 (81)	86 (80)	
Female/male (n)	35/46	36/44	0.82^a^
Age (years) [Mean ± SD (Range)]	51.9 ± 10.9 (25–73)	52.9 ± 10.9 (25–79)	0.72^b^
Course of DM (years) [Mean ± SD (Range)]	11.8 ± 6.4 (0.3–25)	12.3 ± 6.4 (0.5–30)	0.63^b^
Hypertension [n (%)]	47 (54.0%)	51 (59.3%)	0.48^a^
HbA1c (%) [Mean ± SD (Range)]	7.6 ± 1.5 (4.4–12.0)	7.8 ± 1.4 (5.4–10.8)	0.56^b^
eGFR [Mean ± SD (Range)]	70.6 ± 29.4 (7.6–136.9)	71.9 ± 33.0 (4.9–125.6)	0.67^b^

^a^ Chi-squared test for independent variable, ^b^ Mann-Whitney U test. Abbreviations: DM: diabetes mellitus; HbA1c: glycosylated hemoglobin; eGFR: estimated glomerular filtration rate; SD: standard deviation

**Table 1B:** Ocular baseline characteristics and severity

	23-G	25-G	*P*
OD/OS (n)	40/47	38/47	0.88^a^
Baseline BCVA (LogMAR) (Mean ± SD)	1.84 ± 0.97	1.69 ± 0.90	0.29^b^
Baseline IOP (mmHg) (Mean ± SD)	14.7 ± 6.2	14.4 ± 3.9	0.70^b^
CS [Mean ± SD (Range)]	4.03 ± 2.45 (0–8)	4.20 ± 2.47 (0–8)	0.66^b^
FVP grading			
1 [n (%)]	24 (27.6%)	21 (24.4%)	0.78^a^
2 [n (%)]	18 (20.7%)	14 (16.3%)	
3 [n (%)]	32 (36.8%)	37 (43.0%)	
4 [n (%)]	13 (14.9%)	14 (16.3%)	
Underlying Disease			
Iris erythema [n (%)]	1 (1.1%)	1 (1.2%)	> 0.99^c^
NVG	4 (4.6%)	3 (3.5%)	

Abbreviations: LogMAR: logarithm of minimal angle of resolution; IOP: intraocular pressure; CS: preoperative complexity score; FVP: fibrovascular proliferation; NVG: neovascular glaucoma. a Chi-squared test for independent variable, b Mann-Whitney U test, c Fisher’s exact test

**Table 1C:** Ophthalmologic treatment history

	23-gauge	25-gauge	*P*
Previous Ophthalmologic Treatment History			
Retinal photocoagulation [n (%)]	41 (47.13%)	45 (52.33%)	0.49^a^
Anti-VEGF IVI history [> 1month, n (%)]	19 (21.84%)	22 (25.58%)	0.60^a^
Pseudo-phakic/phakic ratio (n)	7/80	10/76	0.46^a^
Pre-OP IVI Anti-VEGF (< 1 month)			
Total [n (%]	57 (65.5%)	55 (64.0%)	0.87^a^
Aflibercept [n (%)]	22 (25.3%)	15 (17.4%)	0.63^a^
Ranibizumab [n (%)]	29 (33.3%)	34 (40.0%)	
Conbercept [n(%)]	6 (6.9%)	6 (7.0%)	
Time of anti-VEGF IVI before PPV (d) [Mean ± SD (Range)]	7.3 ± 5.2 (3–27)	5.7 ± 2.9 (3–23)	0.14^b^

Abbreviations: VEGF, vascular endothelial growth factor; IVI, intravitreal injection; Pre-OP, pre-operative; PPV, Pars plana vitrectomy. Statistical test definitions as in Table 1A

### Intraoperative outcomes

#### General surgical parameters

Intraoperative parameters are summarized in Table 2. The rates of combined phacovitrectomy were comparable between the 23-G and 25-G groups. Similarly, the distribution of tamponade agents (BSS, air, gas, or silicone oil) showed no significant difference (all *P* > 0.05).

**Table 2:** Intraoperative parameters comparison between two groups

	23-G (*n* = 87)	25-G (*n* = 86)	*P*
Phacovitrectomy rate [n (%)]	47 (54.0%)	43 (50.0%)	0.60^a^
Iatrogenic retinal breaks (mean ± SD)	0.31 ± 0.84	0.20 ± 0.68	0.33^b^
Membrane forceps used [n (%)]	22 (25.3%)	16 (18.6%)	0.29^a^
Bimanual techniques used [n (%)]	3 (3.5%)	1 (1.2%)	0.62^a^
Viscodissection [n (%)]	5 (5.8%)	4 (4.7%)	0.75^a^
Intraocular tamponade			
BSS [n (%)]	10 (11.5%)	11 (12.8%)	0.90^a^
Air [n (%)]	50 (57.5%)	52 (60.5%)	
Gas [n (%)]	11 (12.7%)	8 (9.3%)	
SO [n (%)]	16 (18.4%)	15 (17.4%)	
Eyes sutured sclerotomies [n (%)]	73 (83.9%)	20 (23.3%)	< 0.0001^a***^
Operation time			
Total operation time (min) [Mean ± SD (Range)]	63.30 ± 18.38 (33–122)	59.84 ± 19.28 (35–138)	0.14^b^
Vitrectomy time (min) [Mean ± SD (Range)]	36.55 ± 15.54 (16.3–85)	35.59 ± 16.59 (16–116)	0.67^b^
Tool exchanges in vitrectomy			
Total [Mean ± SD (Range)]	2.22 ± 2.71 (0–15)	1.31 ± 2.02 (0–11)	0.003^b**^
In FVP grade 1 (mean ± SD)	0.46 ± 0.58	0.14 ± 0.47	0.031^b*^
In FVP grade 2 (mean ± SD)	1.28 ± 1.37	0.21 ± 0.43	0.002^b**^
In FVP grade 3 (mean ± SD)	3.22 ± 2.93	1.54 ± 1.35	0.0007^b***^
In FVP grade 4 (mean ± SD)	4.31 ± 3.50	3.64 ± 3.39	0.588^b^

Abbreviations: BSS, Balanced Salt Solution, SO, silicone oil, CS, complexity score, FVP, fibrovascular proliferation. Statistical test: a Chi-squared test for independent variable, b Mann-Whitney U test, c Fisher’s exact test, d unpaired t test with Welch correction. *: *p* < 0.05; **: *p* < 0.005; ***: *p* < 0.001

**Fig. 2:**
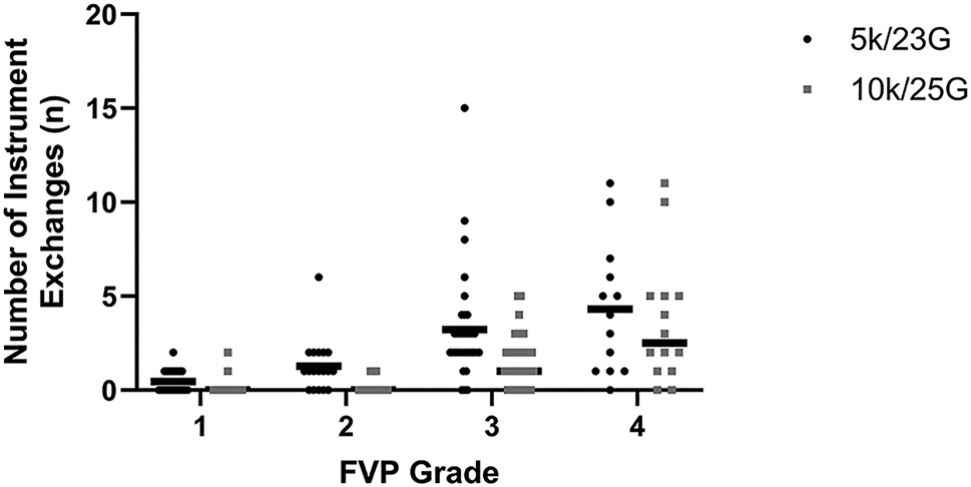
Reduction in instrument exchanges using the 25-G BTP system stratified by FVP Severity. Scatter plots show instrument exchanges in 23-G versus 25-G groups across FVP grades, with the black line indicating the median. *P* < 0.05 for grades 1–3; ns for grade 4. Abbreviations: FVP, fibrovascular proliferation. Data were visualized using GraphPad Prism version 10 (GraphPad Software)

The 25-G BTP system showed significant technical advantages. The number of instrument exchanges during vitrectomy in the 25-G group was reduced by nearly 41% compared to the 23-G group (1.31 ± 2.02 vs. 2.22 ± 2.71, *P* = 0.003). This reduction was statistically significant across FVP grades 1 through 3 (all *P* < 0.05) (Fig. 2). Consequently, the need for sclerotomy suturing was significantly lower in the 25-G group (23.26% vs. 83.91%, *P* < 0.0001).

**Fig. 3:**
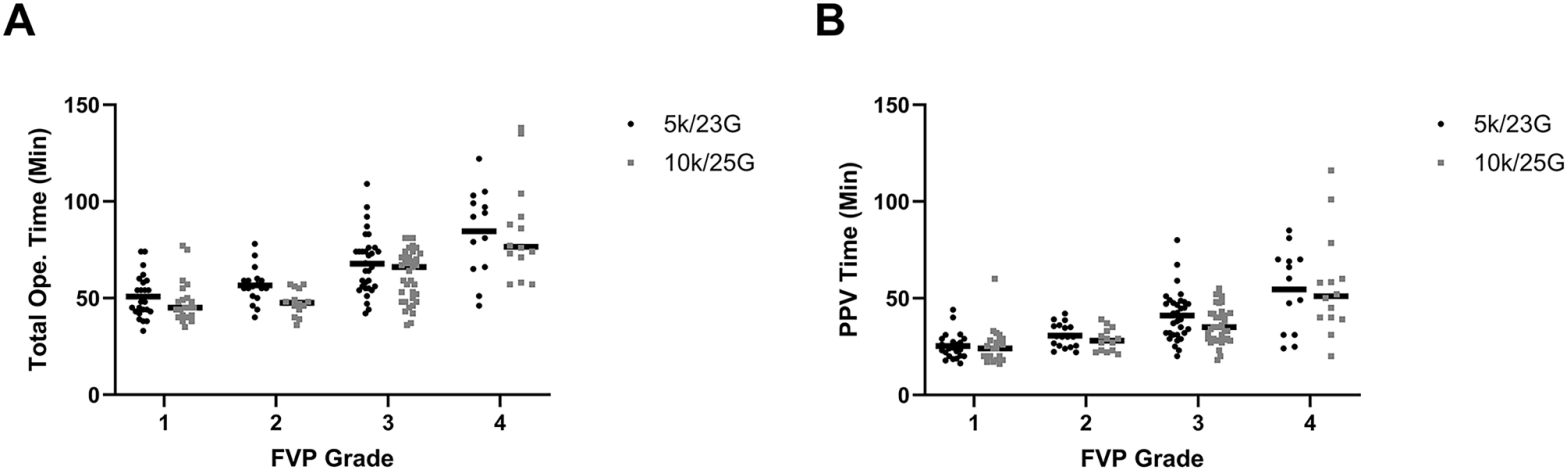
Comparable operative durations between systems across severity grades. Total operation time (**A**) and core PPV time (**B**) by FVP grade (black line indicates the median). There were no significant differences between group at any severity level (all *P* > 0.05). Abbreviations: PPV, pars plana vitrectomy; FVP, fibrovascular proliferation. Generated using GraphPad Prism version 10 (GraphPad Software)

Neither total operation time nor core vitrectomy time differed significantly between groups. These findings were consistent across all FVP grades, with no significant between-group differences observed at any severity level (all *P* > 0.05, Fig. 3). The use of advanced surgical techniques—including membrane forceps, bimanual manipulation and viscodissection techniques—was also similar. The most common complication, iatrogenic retinal breaks, occurred at a low and similar rate (0.20 ± 0.68 vs. 0.31 ± 0.84, *P* = 0.33). No other severe intraoperative complications were observed.

### Endodiathermy utilization for hemostasis

**Fig. 4:**
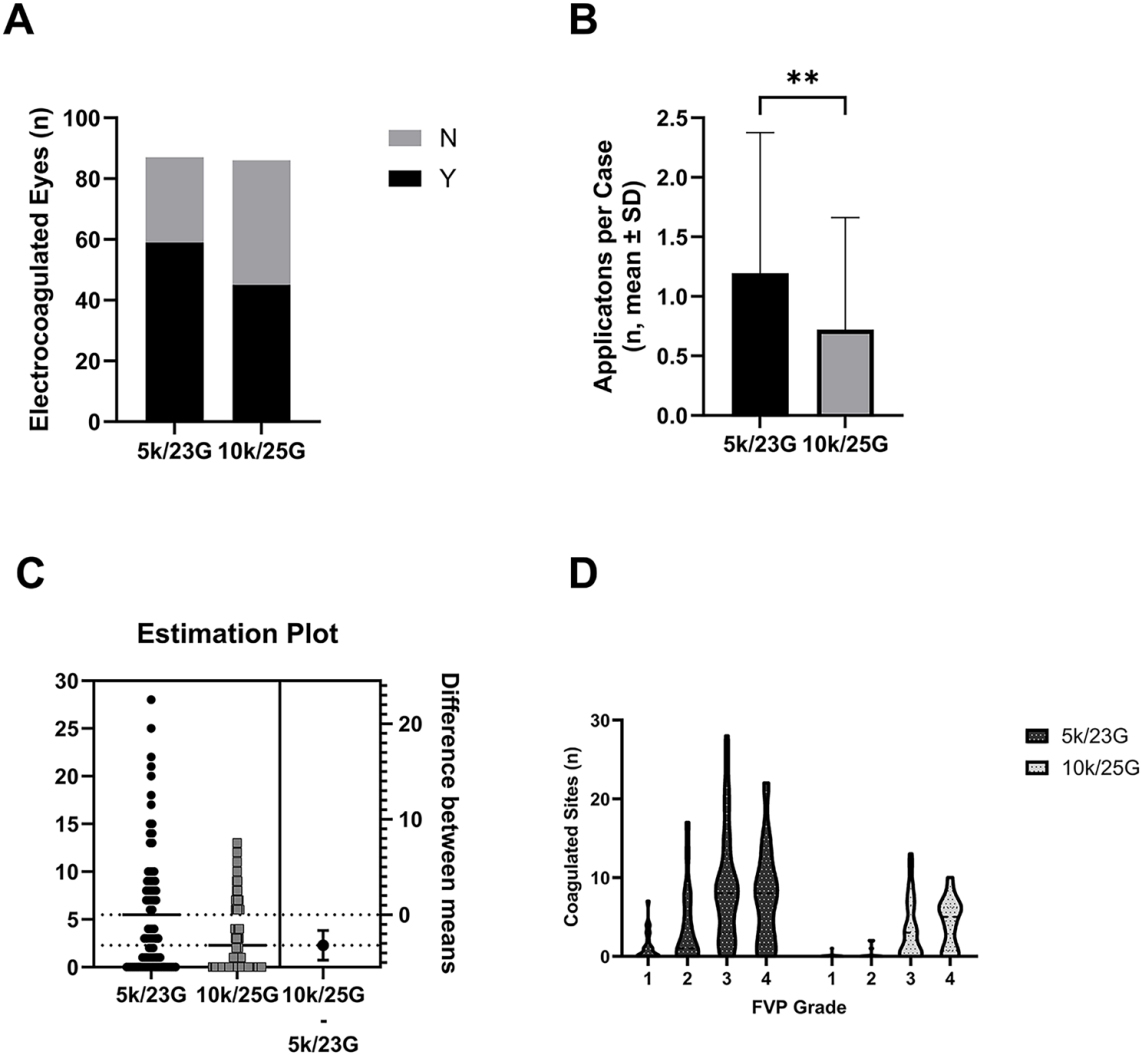
Superior hemostatic control with 25-gauge instrumentation. (**A**) Fewer eyes in the 25-G group required endodiathermy (*P* = 0.04). (**B**) Reduced mean frequency of diathermy application with 25-G (*P* = 0.003). (**C**) Total coagulation sites per eye (25-G vs. 23-G: *P* < 0.0001; estimation plot with mean ± 95% CI). (**D**) Stratified analysis by FVP grade (*P* < 0.05 for grades 1–3; ns for grade 4). Violin plots show median (solid line), IQR (box), and data distribution. Abbreviation: FVP, fibrovascular proliferation. Data analysis and visualization were performed using GraphPad Prism version 10 (GraphPad Software)

Superior hemostatic control was observed with the 25-G BTP system compared to the 23-G FTP approach. Details are provided in Table 3; Fig. 4. Specifically, the advantages include: (1) Reduced Diathermy Requirement: Significantly fewer eyes in the 25-G group required intraoperative endodiathermy (52.3% vs. 67.8%, *P* = 0.04, Fig. 4A). This advantage was particularly pronounced in FVP grades 1–3 (all *P* < 0.05), whereas there was no significant difference observed in grade 4 cases (*P* > 0.99). (2) Reduced Frequency of Diathermy Application: The mean number of diathermy applications per case was nearly halved with 25-G instrumentation (0.72 ± 0.94 vs. 1.20 ± 1.18, *P* = 0.003, Fig. 4B). (3) Markedly Fewer Cauterization Sites: The total number of bleeding sites requiring cauterization was drastically reduced with the 25-G system. In the overall cohort, bleeding sites were 2.28 ± 3.33 with 25-G vs. 5.49 ± 6.49 with 23-G (*P* < 0.0001, Fig. 4C). In the subgroup requiring diathermy, the numbers were 4.92 ± 3.36 vs. 7.83 ± 6.49 sites (*P* = 0.04). This reduction was consistent across FVP grades 1–3 (all *P* ≤ 0.01), but absent in grade 4 cases (*P* = 0.12, Fig. 4D).

**Table 3:** Comparison of endodiathermy for hemostasis between the 23-G and 25-G groups

	23-G	25-G	*P*
Eyes used endo diathermy			
In all eyes [n (%)]	59/87 (67.8%)	45/86 (52.3%)	0.04^a*^
In FVP grade 1 [n (%)]	10/24 (41.67%)	1/21 (4.76%)	0.005^c**^
In FVP grade 2 [n (%)]	10/18 (55.56%)	3/14 (21.43%)	0.08^c^
In FVP grade 3 [n (%)]	29/32 (90.63%)	28/37 (67.57%)	0.04^c*^
In FVP grade 4 [n (%)]	10/13 (76.92%)	11/14 (78.57%)	> 0.999^c^
Frequency of endo diathermy usage			
In all eyes (Mean ± SD)	1.20 ± 1.18	0.72 ± 0.94	0.003^b*^
In electrocoagulation eyes (Mean ± SD)	1.76 ± 1.02	1.56 ± 0.79	0.41^b^
No. Of diathermy sites			
In all eyes (Mean ± SD)	5.49 ± 6.49	2.28 ± 3.33	< 0.0001^b***^
In electrocoagulation eyes			
Total (Mean ± SD)	7.83 ± 6.49	4.92 ± 3.36	0.04^b*^
In FVP grade 1 (mean ± SD)	1.17 ± 1.81	0.05 ± 0.22	0.006^d***^
In FVP grade 2 (mean ± SD)	3.72 ± 5.03	0.36 ± 0.74	0.01^d**^
In FVP grade 3 (mean ± SD)	8.91 ± 7.32	3.54 ± 3.81	0.0005^d***^
In FVP grade 4 (mean ± SD)	7.54 ± 6.55	4.29 ± 3.24	0.12^d^

Abbreviations: SD, standard deviation; FVP, fibrovascular proliferation. Statistical test: a Chi-squared test for independent variable, b Mann-Whitney U test, c Fisher’s exact test, d unpaired t test with Welch correction. *: *p* < 0.05; **: *p* < 0.005; ***: *p* < 0.001

### Postoperative outcomes in 3 months

Early postoperative recovery showed no significant difference between the two groups (Table 4). BCVA gradually improved as the postoperative follow-up time increased. However, BCVA improvement (ΔlogMAR) at 3 months showed no significant difference between groups (-0.91 ± 1.07 vs. -0.89 ± 1.07, *P* = 0.88). Mean IOP remained stable and comparable at all follow-up points, including postoperative day 1, 2 weeks, and 3 months (all *P* > 0.10). Ocular hypertension/hypotony rates were low and statistically equivalent. Serious recurrent VH was rare (23-G: 1 eye at 3 months vs. 25-G: 2 eyes at 1 month; *P* = 0.25 across intervals). Among the two 25-G cases, one resolved spontaneously by month 2. The other case presented at month 3 with a blood-stained cornea, which was attributed to delayed follow-up caused by hospitalization for severe respiratory infection and subsequent discontinuation of treatment. No endophthalmitis, choroidal detachment, or suprachoroidal hemorrhage occurred in either group.

**Table 4:** Comparisons of 3-month postoperative parameters between 23-G FTP and 25-G BTP vitrectomy

	23-G (*n* = 87)	25-G (*n* = 86)	*P*
BCVA improvement at 3 month (logMAR) (mean ± SD)	-0.91 ± 1.07	-0.89 ± 1.07	0.88^b^
Postoperative IOP [mean ± SD (range)]			
1 day	16.11 ± 6.55 (5.8–41.3)	15.60 ± 7.41 (6.2–44.4)	0.27^b^
2 weeks	16.69 ± 7.37 (8–53.6)	15.37 ± 5.03 (8.6–41.8)	0.41^b^
3 months	14.77 ± 3.68 (6.4–29.0)	13.95 ± 4.06 (6.0–31.9)	0.11^b^
Postoperative ocular hypertension / hypotony [n (%)]			
1 day	6 (6.9%) / 1 (1.15%)	6 (6.98%) / 0 (0%)	> 0.999^c^
2 weeks	5 (5.75) / 0 (0%)	3 (3.49%) / 0 (0%)	0.72^c^
3 months	1 (1.15%) / 0 (0%)	1 (1.16%) /0 (0%)	> 0.999^c^
Postoperative Serious VH recurrence [n (%)]			
2 weeks	0 (0%)	0 (0%)	> 0.999^c^
1 month	0 (0%)	2 (2.33%)	0.25^c^
2 months	0 (0%)	0 (0%)	> 0.999^c^
3 months	1 (1.15%)	0 (0%)	> 0.999^c^
Other complications during the postoperative 3 months [n (%)]			
Serious corneal epithelial damage	1 (1.15%)	0 (0%)	> 0.999^c^

Abbreviations: BCVA, best corrected visual acuity; IOP, intraocular pressure; VH, vitreous hemorrhage; NVG, neovascular glaucoma. Statistical test: aChi-squared test for independent variable, bMann-Whitney test, cFisher’s exact test

### Exploratory analysis at 6 months

#### Attrition analysis for 6-month follow-up

Supplementary Table S1 compares patients who completed the 6-month follow-up and those lost to follow-up. Critically, baseline characteristics were well-balanced between the 23-G and 25-G cohorts with completed follow-up (all P₃ >0.05). Notable observations within groups are as follows. In 25-G Group, patients lost to follow-up had lower hypertension rates than completers (41.7% vs. 66.1%, P₂=0.04). In the 23-G Group, completers showed significantly better baseline BCVA (1.7 ± 0.9 vs. 2.1 ± 1.0 logMAR, P₁=0.05). Systemic factors such as age, diabetes duration, HbA1c, and eGFR showed no significant differences between completers and non-completers in either group (all P₁, P₂ >0.10). Operative parameters, including tamponade choice and surgical times, were also comparable across follow-up status groups.

### Functional and structural outcomes

In the 6-month extended cohort (*n* = 126 eyes; 23-G: 64, 25-G: 62), functional and structural outcomes remained comparable between groups. BCVA improvement showed similar vision recovery (-0.99 ± 0.95 vs. -0.80 ± 1.16 logMAR, *P* = 0.36). IOP stability demonstrated equivalent pressure control (14.3 ± 3.5 vs. 13.2 ± 3.9 mmHg, *P* = 0.09). Moreover, complication profiles also showed no significant divergence (Table 5). These exploratory findings suggest sustained outcomes, although statistical power is limited by cohort size and post hoc design.

**Table 5:** Functional outcomes and complications at 6-month follow-up in the exploratory cohort

	23-G (*n* = 64)	25-G (*n* = 62)	*P*
BCVA improvement (logMAR) (mean ± SD)†	−0.99 ± 0.95	−0.80 ± 1.14	0.36^b^
IOP (mean ± SD)	14.3 ± 3.5	13.2 ± 3.9	0.09^b^
Complications [n (%)]§	5 (7.81%)	6 (9.68%)	0.76^c^
Ocular hypertension or NVG [n (%)]	2 (3.13%)	4 (6.45%)	0.44^c^
Recurrent VH [n (%)]	2 (3.13%)	2 (3.23%)	> 0.99^c^
Pre-retinal fibrous proliferative membrane [n (%)]	1 (1.56%)	1 (1.61%)	> 0.99^c^

Exploratory analysis of the 6-month subset (23-G FTP: n = 64; 25-G BTP: *n* = 62). †BCVA improvement: change in logMAR from baseline. §Complications: new-onset events occurring between 3–6 months. Abbreviations: BCVA, best-corrected visual acuity; IOP, intraocular pressure; NVG, neovascular glaucoma; VH, vitreous hemorrhage. Statistical tests: ᵇIndependent t-test or Mann-Whitney U test as appropriate, c Fisher’s exact test (as used in Tables 1, 2, 3 and 4)

### Multivariable analysis results

**Fig. 5:**
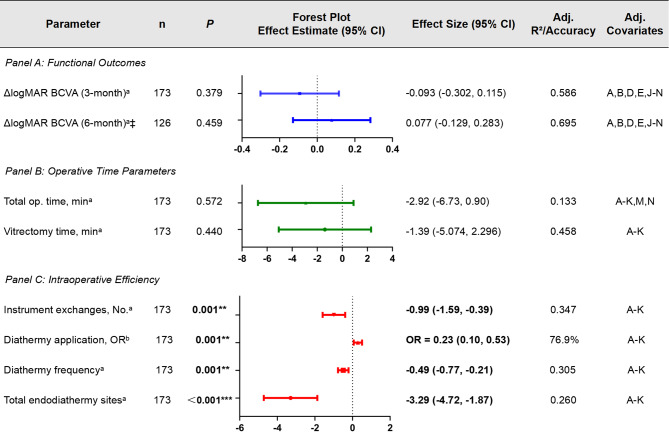
integrated forest plot analysis with multivariable adjusted outcomes comparing 25-G BTP vs. 23-G FTP Systems. All models were adjusted for designated covariates. The following covariate codes were used: A = probe type, B = age, C = sex, D = HbA1c, E = diabetes duration, F = eGFR, G = Scr, H = prior retinal photocoagulation, I = adjuvant anti-VEGF agents, J = FVP grade, K = CS, L = baseline BCVA, M = phacovitrectomy, N = tamponade agent. Filled squares represent point estimates (size proportional to precision). Horizontal lines indicate 95% confidence intervals. The vertical line represents the null effect (β = 0 for continuous outcomes; OR = 1 for diathermy application). ‡6-month follow-up cohort (*n* = 126; follow-up rate: 73.5%). Abbreviations: BCVA, best-corrected visual acuity; op., operation. Statistical tests: aLinear regression (β coefficients) for continuous outcomes; bLogistic regression (OR) for binary outcomes. Adjusted R² is reported for linear models; accuracy for classification model. Statistical significance: **P < 0.005, ***P < 0.001

We performed multivariable regression analyses to isolate the independent effect of the surgical platform (25-G vs. 23-G) on key outcome measures. These analyses rigorously adjusted for a comprehensive set of potential clinical confounders, including age, sex, diabetic control (HbA1c, diabetes duration), renal function (eGFR, Scr), preoperative interventions (retinal photocoagulation, adjuvant anti-VEGF agents), and anatomical complexity (FVP grade, CS score). The models robustly confirmed the superior intraoperative outcomes of the 25-G platform, as visually summarized in Fig. 5. Specifically, the adjusted analyses showed a reduction of 3.29 diathermy sites (95% CI: -4.72 to -1.87; *P* < 0.001), 0.99 fewer instrument exchanges (95% CI: -1.59 to -0.39; *P* = 0.001), and a 77.2% lower odds of diathermy use (OR = 0.228; 95% CI: 0.10–0.53; *P* = 0.001). The similar effect sizes between unadjusted and fully adjusted models confirm the robustness of these findings.

Beyond the primary instrument comparison, our models identified several significant independent predictors for specific outcomes. For clarity, only statistically significant results (*P* < 0.05) are presented here and in Supplementary Table 2. Anatomical Severity Impacts: FVP grading independently increased diathermy requirements by 1.63 sites per grade (*P* = 0.02). The CS substantially prolonged PPV time by 4.26 min per point (*P* < 0.001) and increased instrument exchange frequency by 0.53 exchanges per point (*P* < 0.001). Clinical Decision Modulators: Tamponade agent selection significantly affected total operative time and 6-month BCVA improvement (all *P* < 0.05). Combined phacoemulsification and IOL implantation increased total surgical duration by 7.73 min (*P* = 0.001). Baseline-Driven Visual Recovery: Baseline BCVA was the strongest predictor of postoperative visual gains at both 3 months (B = -0.857; *P* < 0.001) and 6 months (B = -0.970; *P* < 0.001).

In summary, while the 25-G platform’s advantages in hemorrhage control and operational efficiency remain robust after multivariate adjustment, this analysis comprehensively contextualizes outcomes within their anatomical and clinical drivers. This demonstrates that the technical superiority of the 25-G BTP platform and the Trim-and-Excision Technique works together with, rather than independently of, key disease and treatment parameters.

## Discussion

The proliferative diabetic vitreoretinal interface presents formidable surgical challenges. These challenges arise from multiple dense adhesions between the ischemic retina and fibrovascular membrane, which is embedded within the altered posterior hyaloid cortex. Additionally, fragile neovascular pegs further complicate the condition. Besides conventional segmentation and delamination, advanced techniques–including bimanual techniques, trimanual vitrectomy [10] and viscodissection [11]--facilitate membrane removal in complex cases; however, they inherently rely on ancillary instrumentation. The advent of MIVS brought a transformative technique called the all-probe lift and shave [4], which significantly reduced the need for ancillary tool exchanges during fibrovascular dissection. However, a key limitation of this technique is the risk of excessive hemorrhage and iatrogenic retinal tears if dissection is too aggressive [12]. Such complications often require frequent diathermy and can make surgery more difficult.

The evolution toward MIVS with 23-G, 25-G, and 27-G platforms has brought well-documented benefits, including minimized sclerotomy tissue damage, reduced post-operative hypotony and inflammation [13–15]. Consequently, the adoption of these platforms has expanded across a broad range of vitreoretinal pathologies [16]. Since its introduction, 25-G vitrectomy has been extensively utilized for a multitude of surgical indications. The smaller radius of 25-G tubing, combined with high cutting rates and valved cannulas, ensures a stable and controlled dissection of membranes with minimal movement of the underlying retina [17]. Therefore, it reduces the risk of iatrogenic retinal breaks [18]. However, a significant dichotomy persists in clinical practice. Despite these general advantages, many vitreoretinal surgeons maintain a preference for 23-G instrumentation in complex surgical cases, such as advanced PDR. This skepticism is rooted in evidence suggesting enhanced instrument rigidity, efficient fluidics, superior illumination, and a more versatile arsenal of instruments with 23-G systems. To date, despite the growing adoption of microincision systems, only a handful of studies have specifically examined bevelled-tip MIVS designs in vitreoretinal surgery [5, 19–21]. Our investigation addresses this gap by focusing on the intraoperative precision of the 10k/25-G BTP system. This system embodies three pivotal design innovations compared to conventional 5k/23-G FTP systems: gauge reduction (25-G vs. 23-G), tip geometry (beveled-tip vs. flat-tip), and cutting technology (10k rpm vs. 5k rpm). The study was conducted in day surgery for PDR.

Our findings provide robust evidence to resolve the long-standing clinical debate. Critically, we confirmed that there were no statistically significant differences in total operative duration (*P* = 0.14) or vitrectomy time (*P* = 0.67) (Table 2). These results show that the theoretical advantages of 23-G instrumentation do not lead to faster surgery in this complex context. More importantly, the data reveal a decisive superiority of the 25-G BTP system in surgical precision and safety, demonstrated by a 41% reduction in instrument exchanges (1.31 ± 2.02 vs. 2.22 ± 2.71, *P* = 0.003), a 58% decrease in endodiathermy sites (2.28 ± 3.33 vs. 5.49 ± 6.49, *P* < 0.0001), and a 15% lower rate of diathermy application (52.3% vs. 67.8%, *P* = 0.04) (Table 3). Crucially, multivariate analysis was performed considering anatomical complexity (FVP grade), diabetic control (HbA1c), and preoperative interventions. This analysis confirmed that the advantages observed are intrinsic to the 25-G platform (Electrocoagulation sites: B = -3.29, *P* < 0.001; Instrument exchanges: B = -0.99, *P* = 0.001; Diathermy utilization: OR = 0.23, *P* = 0.001). This establishes the 25-G BTP’s technical superiority as independent of confounding anatomical or metabolic factors. Particularly noteworthy is its maintained advantage across progressive FVP grades—a significant finding given these cases typically require more extensive membrane dissection and inherently carry greater hemorrhagic risk (FVP-adjusted B = 1.63 for diathermy sites, *P* = 0.020). The instruments demonstrated remarkable resilience in high-complexity scenarios where conventional MIVS limitations would theoretically manifest most severely. This trifecta of design innovations in the 25-G BTP—gauge reduction, beveled-tip geometry, and 10k-RPM cutting—synergistically empowers it to overcome the traditional limitations of MIVS in PDR surgery. This leads to a paradigm shift, enabling safer and more controlled dissection of fibrovascular membranes while significantly reducing intraoperative hemorrhage, as definitively shown by our data. This confirms the foundational work on 25-G systems by Fujii et al. [22] while decisively extending its applicability and validating its superiority in managing complex PDR.

Crucially, our surgical observations provide real-world context to these metrics. Regarding instrument utilization, vitrectors alone effectively removed most fibrovascular proliferation in both groups. Residual peripapillary membranes needed additional membrane forceps manipulation, while advanced bimanual or viscodissection techniques were rarely used (< 5% of cases). Regarding postoperative safety, reassuringly, no significant differences were observed in key safety endpoints—including intraocular pressure control, surgical success rates, and vision-threatening complications (such as vitreous hemorrhage recurrence) at 3 and 6 months (all *P* >0.05). This aligns with prior studies confirming gauge-independent safety [23, 24]. Patient Comfort: A key difference was observed in suture requirements—significantly lower with the 25-G system (23.3% vs. 83.9%, *P* < 0.0001). This leads directly to better postoperative comfort and reduced risk of astigmatism, representing a clear patient benefit from the smaller gauge size. Functional Recovery Equivalence: Importantly, both systems showed similar visual rehabilitation trajectories. BCVA improvements at 3 months were − 0.91 ± 1.07 versus − 0.89 ± 1.07 (*P* = 0.88), and at 6 months, -0.99 ± 0.95 versus − 0.80 ± 1.14 (*P* = 0.36). Multivariate models confirmed this equivalence stemmed from baseline visual acuity rather than instrumentation (3 M: B = -0.857, *P* < 0.001; 6 M: B = -0.970, *P* < 0.001), with tamponade choice emerging as the only modifiable surgical factor influencing 6-month outcomes (B = 0.191, *P* = 0.031). Taken together, these findings demonstrate that the 25-G BTP system offers technical precision without compromising safety, while providing clear patient benefits. This foundation supports further exploration into the mechanisms behind its efficacy.

The 25-G BTP cohort showed a markedly reduced reliance on endodiathermy, directly indicating a fundamental decrease in intraoperative hemorrhage. This hemostatic superiority, scarcely detailed in prior PDR literature, stems from a confluence of engineered design and adapted surgical technique. First, we can confidently exclude IOP fluctuations as a confounding factor, as the Constellation platform’s dynamic control system ensures equivalent IOP stability across gauge sizes [25]. The observed bleeding reduction is therefore intrinsically linked to the 25-G BTP instrument itself. Most critically, the beveled-tip design confers versatile intraocular maneuverability, integrating multifunctions into a single probe: tissue elevation (pick-like lifting), aspiration-assisted delamination (forceps-mimicking peeling), blunt dissection (viscoelastic-like tissue separation using the beveled edge), and precision segmentation (scissor-equivalent cutting within epiretinal vascular bridge crevices). This not only reduces the need for ancillary instrumentation [3] but also provides a safer and more delicate dissection of abnormal posterior hyaloid and fibrovascular membranes directly. This technological evolution facilitates our refined *Trim and Excision* Technique: Precision Trimming: The beveled tip allows for safe, circumferential trimming of fibrovascular pegs at their base under direct visualization, minimizing traction on adherent vessels. Controlled Excision: Segmented membranes are then excised with high-speed cutting, negating the need for dangerous peeling or lifting maneuvers that stretch and avulse fragile neovascular complexes. Although meticulous, this technique ultimately saves time by preempting iatrogenic vascular breaks and their subsequent management. It is this synergy—a purpose-designed instrument enabling a safer surgical strategy—that underpins our findings of reduced bleeding, fewer instrument exchanges, and equivalent operative times. The multivariate models further validated this synergy, demonstrating that connective tissue strength (CS score)—not gauge selection—dictated PPV duration (B = 4.26/min, *P* < 0.001), indirectly confirming the efficiency of our technique even in anatomically challenging eyes. The reduction in intraoperative hemorrhage and the need for electrocoagulation are undoubtedly significant for protecting the extremely weakened retina and retinal blood vessels. In our cohort, the incidence of both early (≤ 2 week) and recurrent postoperative vitreous hemorrhage (PVH) was comparably low between groups and markedly lower than historical PDR vitrectomy reports [26, 27]; this likely reflects contemporary advancements including routine preoperative anti-VEGF use and the reduced sclerotomy trauma inherent to MIVS, which mitigates entry-site neovascularization.

Although our data confirm that BCVA recovery does not depend on gauge size, interpreting these biological results requires considering the limitations of the available measurement methods. The lack of functional assessments beyond BCVA (such as microperimetry, contrast sensitivity, or reading speed) restricts our ability to identify subtle neuroadaptive benefits from advanced surgical techniques.

## Limitations and future directions

This research has inherent limitations. First, our study uses a hybrid design that includes a prospective 25-G BTP cohort and a retrospective 23-G FTP control group. This design inherently poses a higher risk of selection bias compared to a randomized controlled trial. To reduce these risks, all cases were consecutively enrolled, and baseline characteristics—including validated PDR severity gradings (FVP and CS)—were carefully matched (Table 1), ensuring balanced comparisons for the measured outcomes. Furthermore, all surgical procedures were performed by a single high-volume surgeon who has conducted over 3,000 PPV procedures in the past decade, nearly half of which related to PDR. This extensive experience drastically reduced technical variability, which is a noted strength of the study. Second, the 3-month follow-up period, while sufficient to capture our primary endpoints of intraoperative efficiency and short-term safety, is inadequate to evaluate long-term complications and anatomical stability. However, we conducted supplementary retrospective tracking of both cohorts extending to 6 months postoperatively. Reassuringly, this extended data confirmed: stable anatomical outcomes, persistent functional equivalence: maintained BCVA improvement, and low complication rates. This extended observation, though non-protocolized, substantiates the durability of our core findings within the studied timeframe. Finally, the single-center design enhances internal validity but limits generalizability. Future studies should include longer follow-up periods of 6 to 12 months and adopt a fully prospective, randomized, multicenter design. They should also utilize advanced functional metrics, such as microperimetry and quality-of-life questionnaires, to confirm our findings and investigate more subtle neurosensory effects.

## Conclusions

In conclusion, both 23-G FTP and 25-G BTP systems achieve similar visual and anatomic outcomes in PDR day surgery. However, the 25-G BTP probe improves surgical precision through its integrated design, combining gauge miniaturization, beveled-tip geometry, and 10k-RPM cutting dynamics. This engineering triad enables the novel *Trim and Excision* Technique, which allows targeted dissection of fibrovascular membranes and vitreoretinal adhesions while minimizing vascular injury. Clinically, this translates to a paradigm shift: (1) Substantially reduced dependence on ancillary instrumentation; (2) Drastically decreased intraoperative hemorrhage and subsequent diathermy demand; (3) Preservation of retinal structural integrity. Collectively, these advantages position 25-G BTP as a safer and more efficient surgical platform for complex PDR cases, advancing the standard of microincisional vitreoretinal surgery.

## Electronic supplementary material

Below is the link to the electronic supplementary material.

Supplementary Material 1

Supplementary Material 2

## Data Availability

The clinical data supporting this study are available from Xi’an People’s Hospital (Xi’an Fourth Hospital) Ethics Committee but restrictions apply to the availability of these data. Data are however available from the authors upon reasonable request and with permission of the ethics committee.

## Abbreviations

AMD: Macular degeneration
BCVA: Best corrected visual acuity
BSS: Balanced Salt Solution
BTP: Beveled-tip probe
cpm: Cuts per minutes
CS: Preoperative complexity score
DAP: Diastolic arterial pressure
DM: Diabetes mellitus
eGFR: Estimated glomerular filtration rate
FTP: Flat-tip probe
FVP: Fibrovascular proliferation
HbA1c: Glycosylated hemoglobin
IOP: Intraocular pressure
IVI: Intravitreal injection
LogMAR: Logarithm of minimal angle of resolution
MIVS: Micro-incisional vitrectomy system
NVG: Neovascular glaucoma
PDR: Proliferative diabetic retinopathy
PPV: Pars plana vitrectomy
Pre-OP: Pre-operative
PRP: Pan-retinal photocoagulation
PVD: Posterior vitreous detachment
RD: Retinal detachment
SAP: Systolic arterial pressure
Scr: Serum creatinine
SD: Standard deviation
SO: Silicon oil
SS-OCT: Swept-source optical coherence tomography
TRD: Tractional retinal detachment
VEGF: Vascular endothelial growth factor
VH: Vitreous hemorrhage
