# Correction: Intraoperative precision of 25-gauge beveled-tip versus 23-gauge flat-tip probes in day surgery vitrectomy for proliferative diabetic retinopathy: a comparative cohort study

**DOI:** 10.1186/s40942-025-00780-4

**Published:** 2025-12-22

**Authors:** Daxi Xue, Yanchun Zhang, Ziwei Kang, Jiamin Zheng, Yingnan He, Xin Luo

**Affiliations:** Shaanxi Eye Hospital, Xi’an People’s Hospital (Xi’an Fourth Hospital), Affiliated People’s Hospital of Northwest University, No. 21 Jiefang Road, Xi’an, Shaanxi 710004 China

**Correction: International Journal of Retina and Vitreous (2025) 11:123**

10.1186/s40942-025-00746-6

The abstract for this article was inadvertently published with error. In the sentence beginning ‘Sutureless sclerotomy rates were higher in the 23-G group (83.9% vs. 23.3%, *P* < 0.0001).’ should have read as ‘Sutureless sclerotomy rates were higher in the 25-G group (76.7% vs. 16.1%, *P* < 0.0001)’.

The original article has been corrected

