# Supplementary material for: Intraoperative precision of 25-gauge beveled-tip versus 23-gauge flat-tip probes in day surgery vitrectomy for proliferative diabetic retinopathy: a comparative cohort study
Source: Int J Retina Vitreous. 2025 Nov 5;11:123. doi: 10.1186/s40942-025-00746-6 (PMC12587617; doi:10.1186/s40942-025-00746-6)
Supplement: Supplementary file 1 — Supplementary Material 1 [file 40942_2025_746_MOESM1_ESM.docx]

**Supplementary Table S1: Baseline Comparison Between Patients Completing and Lost to 6-Month Follow-up**

| **Variable** | **23G (n=87)** | | | **25G (n=86)** | | | ***P3*** |
| --- | --- | --- | --- | --- | --- | --- | --- |
|  | **Followed (n=64)** | **Lost (n=23)** | ***P1*** | **Followed (n=62)** | **Lost (n=24)** | ***P2*** |  |
| Female/male (n) | 27/37 | 10/13 | 0.91^a^ | 30/32 | 7/17 | 0.11^a^ | 0.48^a^ |
| Age (years) [Mean ± SD] | 51.2 ± 10.7 | 54.1 ± 11.4 | 0.29^b^ | 51.8 ± 11.2 | 55.5 ± 9.8 | 0.32^b^ | 0.73^b^ |
| Course of DM (years) [Mean ± SD] | 11.3 ± 6.3 | 13.5 ± 6.7 | 0.16^b^ | 11.9 ± 5.9 | 13.5 ± 7.5 | 0.31^b^ | 0.53^b^ |
| Hypertension [n (%)] | 34 (53.1) | 13 (56.5) | 0.78^a^ | 41 (66.1) | 10 (41.7) | **0.04**^a*^ | 0.14^a^ |
| HbA1c(%) [Mean ± SD] | 7.5 ± 1.3 | 7.8 ± 1.9 | 0.71^b^ | 7.6 ± 1.4 | 8.0 ± 1.5 | 0.13^b^ | 0.85^b^ |
| eGFR [Mean ± SD] | 73.1 ± 31.2 | 66.6 ± 27.8 | 0.23^b^ | 69.4 ± 33.3 | 79.7 ± 29.1 | 0.15^b^ | 0.58^b^ |
| OD/OS (n) | 26/38 | 14/9 | 0.09^a^ | 28/34 | 10/14 | 0.77^a^ | 0.61^a^ |
| Baseline BCVA (LogMAR）[Mean ± SD] | 1.7 ± 0.9 | 2.1 ± 1.0 | 0.05^b^ | 1.6 ± 0.9 | 1.9 ± 0.9 | 0.14^b^ | 0.35^b^ |
| Baseline IOP (mmHg) [Mean ± SD] | 14.7 ± 5.6 | 14.7 ± 7.9 | 0.30^b^ | 14.6 ± 4.1 | 13.8 ± 3.4 | 0.46^b^ | 0.75^b^ |
| Previous PRP [n (%)] | 33 (51.6) | 8 (34.8) | 0.17^a^ | 29 (46.8) | 12 (50.0) | 0.79^a^ | 0.59^a^ |
| Pseudo-phakic/phakic ratio (n) | 7/57 | 0/23 | 0.18^c^ | 6/56 | 4/20 | 0.46^c^ | >0.99^c^ |
| Pre-OP IVI Anti-VEGF (<1 month)， n (%) | 43 (67.2) | 14 (60.9) | 0.62^c^ | 39 (62.9) | 16 (66.7) | 0.81^c^ | 0.71^c^ |
| Time of anti-VEGF IVI before PPV (d) [Mean ± SD] | 6.7 ± 4.2 | 8.9 ± 7.5 | 0.51^b^ | 5.8 ± 3.3 | 5.4 ± 1.4 | 0.60^b^ | 0.24^b^ |
| CS [Mean ± SD] | 4.3 ± 2.5 | 3.3 ± 2.3 | 0.07^b^ | 4.5 ± 2.6 | 3.4 ± 2.0 | 0.06^b^ | 0.66^b^ |
| FVP grading：1/2/3/4 (n) | 13/14/27/10 | 11/4/5/3 | 0.08^a^ | 13/9/28/12 | 8/5/9/2 | 0.39^a^ | 0.74^a^ |
| iris erythema and NVG [n (%)] | 3 (4.7) | 2 (8.7) | 0.62^c^ | 3 (4.8) | 1 (4.2) | >0.99^c^ | >0.99^c^ |
| Phacovitrectomy rate [n (%)] | 31 (48.4) | 16 (69.6) | 0.08^a^ | 33 (53.2) | 10 (41.7) | 0.34^a^ | 0.59^a^ |
| Intraocular tamponade: BSS/Air/Gas/SO (n) | 6/36/8/14 | 4/14/3/2 | 0.46^a^ | 8/33/7/14 | 3/19/1/1 | 0.1^a^ | 0.93^a^ |
| Total operation time (min) (Mean ± SD) | 63.7 ± 19.5 | 62.3 ± 15.3 | 0.91^b^ | 62.3 ± 20.6 | 53.5 ± 13.8 | 0.07^b^ | 0.60^b^ |
| Vitrectomy time (sec) (Mean ± SD) | 38.0 ± 16.4 | 32.5 ± 12.1 | 0.67^b^ | 37.0 ± 18.2 | 31.8 ± 11.0 | 0.22^b^ | 0.67^b^ |

P₁: Within-group comparison (23-G Completers vs. 23-G Lost), P₂: Within-group comparison (25-G Completers vs. 25-G Lost), P₃: Between-group comparison (23-G Completers vs. 25-G Completers). Abbreviations: DM, diabetes mellitus; eGFR, estimated glomerular filtration rate; BCVA, best-corrected visual acuity; LogMAR, logarithm of minimal angle of resolution; IOP, intraocular pressure; PRP, pan-retinal photocoagulation; Pre-OP, pre-operative; IVI, intravitreous injection; PPV, pars plana vitrectomy; CS, preoperative complexity score; FVP, fibrovascular proliferation; NVG, neovascular glaucoma; BSS, Balanced Salt Solution; SO, silicone oil. Statistical tests: ^a^ Chi-squared test; ^b^ Mann-Whitney test; ^c^ Fisher’s exact test.*P < 0.05.
