# Supplementary material for: Intraoperative precision of 25-gauge beveled-tip versus 23-gauge flat-tip probes in day surgery vitrectomy for proliferative diabetic retinopathy: a comparative cohort study
Source: Int J Retina Vitreous. 2025 Nov 5;11:123. doi: 10.1186/s40942-025-00746-6 (PMC12587617; doi:10.1186/s40942-025-00746-6)
Supplement: Supplementary file 2 — Supplementary Material 2 [file 40942_2025_746_MOESM2_ESM.docx]

**Supplementary Table S2: Significant Predictors from Multivariable Surgical Outcome Analyses**

| **Variable** | **Effect Size (95% CI)** | **P Value** | **Adj R^2^/Accuracy** | **Adj. Covariates^*^** |
| --- | --- | --- | --- | --- |
| Diathermy application, OR (n = 173)^b^ | | | | |
| 25-G (vs. 23-G) | 0.23 (0.10, 0.53) | 0.001 | 76.9% | A-K |
| CS Score | 1.63 (1.18, 2.27) | 0.003 |  |  |
| Total endodiathermy sites (n = 173)^a^ | | | | |
| 25-G (vs. 23-G) | -3.29 (-4.72,-1.87) | ＜0.001 | 0.26 | A-K |
| FVP Grade | 1.63 (0.26, 3.00) | 0.020 |  |  |
| Diathermy frequency (n = 173)^a^ | | | | |
| 25-G (vs. 23-G) | -0.49 (-0.77, -0.21) | 0.001 | 0.31 | A-K |
| CS Score | 0.16 (0.04, 0.27) | 0.01 |  |  |
| Total instrument exchanges (n = 173)^a^ | | | | |
| 25-G (vs. 23-G) | -0.99 (-1.59, -0.39) | 0.001 | 0.35 | A-K |
| CS Score | 0.53 (0.28 to 0.78) | <0.001 |  |  |
| Total operative time (min, n = 173)^a^ | | | | |
| CS Score | 3.07 (1.29, 4.84) | 0.001 | 0.57 | A-K, M, N |
| Combined phaco and IOL | 7.73 (3.33, 12.13) | 0.001 |  |  |
| Tamponade agent | 7.14 (4.04, 10.24) | 0.001 |  |  |
| Vitrectomy time (min, n = 173)^a^ | | | | |
| CS Score | 4.26 (2.70, 5.816) | <0.001 | 0.44 | A-K |
| BCVA Improvement(3-mo), ΔlogMAR (n = 173)^a^ | | | | |
| Baseline BCVA, logMAR | -0.86 (-0.97, -0.75) | <0.001 | 0.59 | A, B, D,E, J-N |
| BCVA Improvement (6-mo), ΔlogMAR (n = 126)^a^‡ | | | | |
| Baseline BCVA, logMAR | -0.97 (-1.08, -0.86) | <0.001 | 0.72 | A, B, D,E, J-N |
| Tamponade agent | 0.19 (0.02, 0.36) | 0.03 |  |  |

All models adjusted for designated covariates. *Covariate codes: A = Probe type; B = Age; C = Sex; D = HbA1c; E = Diabetes duration; F = eGFR; G = Serum creatinine (Scr); H = Prior retinal photocoagulation; I = Adjuvant anti-VEGF agents; J = FVP grade; K = CS score; L = Baseline BCVA; M = Combined phaco and IOL with vitrectomy; N = Tamponade agent. ^‡^Complete 6 months case analysis (follow-up rate: 73.5%). Abbreviations: OR, Odds Ratio; CS: preoperative complexity score; FVP: fibrovascular proliferation; IOL, intraocular lens; BCVA, best-corrected visual acuity; LogMAR: logarithm of minimal angle of resolution. Statistical tests: ^a^Linear regression (β coefficients) for continuous outcomes， ^b^Binary outcomes: logistic regression (OR) for binary endpoints. Adjusted R² reported for linear models; Accuracy reported for classification models.
